# Supplementary material for: A systematic review and meta-analysis of thigmotactic behaviour in the open field test in rodent models associated with persistent pain
Source: PLoS One. 2023 Sep 8;18(9):e0290382. doi: 10.1371/journal.pone.0290382 (PMC10490990; doi:10.1371/journal.pone.0290382)
Supplement: S1 File — (DOCX) [file pone.0290382.s001.docx]

# S1: Summary Tables of Acclimitisation, Animal Husbandry and Experimental Conditions

**Table 1**. Out of the 181 included studies, the number of studies which reported information on acclimatisation and animal husbandry

| **Criterion** | **Number of studies reported** | **Range and median** |
| --- | --- | --- |
| Animals given a time period to acclimatise | 75 | Median = 168 hr;  Range = 1 – 504 hr |
| Number of animals housed per cage | 77 | Median = 3  Range = 1 – 8 |
| Different animal sexes housed in the same room | 5 | N/A |
| Different animal species housed in the same room | 1 | N/A |
| Size of the living cage | 5 | N/A |
| Living cages covered by bedding materials | 19 | N/A |
| Temperature of the housing environment | 113 | Range = 19 – 27°C  Median = 22°C; |
| Humidity of the housing environment | 59 | Range = 30 – 60%  Median = 50%; |
| Noise level of the housing environment | 0 | N/A |
| Vibration level of the housing environment | 0 | N/A |
| Number of light/dark cycle | 153 | Range = 12/12 hr – 14/10 hr  Median = 12/12 hr; |

**Table 2**. Out of the 181 included studies, the number of studies which reported information on experimental conditions

| **Criterion** | **Number of studies reported** | **Range and median** |
| --- | --- | --- |
| OFT conducted in an isolated chamber | 19 | N/A |
| Test arena light intensity | 52  (51 studies reported in lux; 1 study reported in watts) | Range = 0 – 1000 lux  Median = 30 lux  Reported at = 40 w |
| Temperature in the testing room | 6 | Range = 22 – 25°C  Median = 22°C |
| Humidity in the testing room | 2 | Range = 30 – 45%  Median = 38% |
| Noise level in the testing room | 5 | Range = 40 – 65 dB  Median = 40 dB |
| Vibration level of the testing room | 0 | N/A |
| Height of the open arena | 128 | Range = 15 – 100cm  Median = 40cm |
| Shape of the open arena | 173 | Circle = 11 studies  Rectangular/Square = 162 studies |
| Shape of the inner zone | 110 | Circle = 9 studies  Rectangular/Square = 101 studies |
| Total area of the open arena | 170 | Range = 100 – 1000000cm^2^  Median = 2500cm^2^ |
| Total area of the inner zone | 90 | Range = 40 – 12337cm^2^  Median = 900cm^2^ |
| Location of where the animal was placed at the start of the test | 120 | N/A |
| Method of measurement | 135 | Automated measurement = 128 studies  Manual measurement = 7 studies |
