# Supplementary material for: A systematic review and meta-analysis of thigmotactic behaviour in the open field test in rodent models associated with persistent pain
Source: PLoS One. 2023 Sep 8;18(9):e0290382. doi: 10.1371/journal.pone.0290382 (PMC10490990; doi:10.1371/journal.pone.0290382)
Supplement: S2 File — (DOCX) [file pone.0290382.s002.docx]

# S2: Analysis of Experiments Using Naïve Controls

**Rat modelling experiments using naïve controls**

***Thigmotaxis was increased by injury and disease models associated with persistent pain compared to naïve controls in rats***

A total of 10 studies, containing 12 cohort-level comparisons, 165 rats, and a N range from 6 to 24 with a median of 13 per group, assessed the effects of 7 types of disease models associated with persistent pain on thigmotaxis in 4 rat strains. Sprague-Dawley and Wistar rats were the most reported strains (both 42%, k = 5). Male rats were used in 75% (k = 9) of experiments and female rats were used in 25% (k = 3). Eighty-three percent (k = 10) of experiments assessed thigmotaxis by measuring time spent in the centre, and 17% (k = 2) measured number of central crossings.

Overall, the disease models significantly increased thigmotaxis when compared to the naïve controls (SMD = -2.86 [95% CI -3.90 to -1.83]). Heterogeneity was high (Q = 77.10, df = 11, *p* < 0.0001, *I^2^* = 86%) (Figure).

Figure. A summary forest plot of the 12 cohort-level comparisons which assessed the impact of modelling on thigmotaxis in rats (using naïve controls). For each comparison, an effect size was calculated using the Hedges’ g SMD method. Effect sizes were pooled using the random effects model. The restricted maximum-likelihood method was used to estimate heterogeneity. The overall effect size is -2.86 [95% CI -3.90 to -1.83]; Q = 77.10, *df* = 11, *p* < 0.0001, *I^2^* = 86%. The size of the square represents the weight, which reflects the contribution of each comparison with the pooled effect estimate. CI, confidence interval; N, number of animals.

Stratified meta-analysis is restricted by the insufficient number of cohort-level comparisons for each characteristic, therefore we cannot investigate the influence of study characteristics on thigmotactic outcomes.

**Mouse modelling experiments using naïve controls**

***Limited data could not determine the magnitude of how thigmotaxis was affected by injury and disease models associated with persistent pain in mouse experiments using naïve controls***

A total of 3 studies, containing 5 cohort-level comparisons, 88 mice, and a N range from 12 to 32 with a median of 16, assessed the effects of 4 types of disease models associated with persistent pain on thigmotaxis in C57BL/6 rat strain. Male rats were used in 40% (k = 2) of experiments and female rats were used in 60% (k = 3). All experiments assessed thigmotaxis by measuring time spent in the centre. A meta-analysis is restricted by the insufficient number of cohort-level comparisons.
