# Supplementary material for: A systematic review and meta-analysis of thigmotactic behaviour in the open field test in rodent models associated with persistent pain
Source: PLoS One. 2023 Sep 8;18(9):e0290382. doi: 10.1371/journal.pone.0290382 (PMC10490990; doi:10.1371/journal.pone.0290382)
Supplement: S3 File — (DOCX) [file pone.0290382.s003.docx]

# S3: Effects of Experimental Conditions and Apparatus on Thigmotactic Outcomes

**Table S3.1. Meta-regressions of the effects of the experimental conditions and apparatus on thigmotactic outcome in rat and mouse experiments**

| **Experiment type** | **Dataset** | **Variable** | **Comparisons (k)** | **Significance of the regression model** |
| --- | --- | --- | --- | --- |
| **Rat modelling** | 1 | Colour of the testing arena wall | • Black (k = 18)  • Transparent (k = 12) | *p* = 0.99 |
|  | 1 | Location of where the animal was placed in the arena | • Centre (k = 51)  • Corner (k = 12) | *p* = 0.48 |
|  | 1 | OFT assessment duration | • 5 mins (k = 37)  • 10 mins (k = 29)  • 15 mins (k = 14) | *p* = 0.13 |
| **Rat analgesic drug treatment** | 2 | Height of the wall of the open field arena | • 50 cm (k = 15)  • 40 cm (k = 14) | *p* = 0.21 |
| **Mouse modelling** | 3 | Height of the wall of the open field arena | • 40 cm (k = 40)  • 30 cm (k = 11) | *p* = 0.09 |
|  | 3 | Total area of the open arena | • 1600 cm^2^ (k = 53)  • 2500 cm^2^ (k = 27) | *p* = 0.15 |
|  | 3 | Total area of the inner zone | • 1296 cm^2^ (k = 24)  • 400 cm^2^ (k = 22) | *p* = 0.004 |
|  | 3 | Location of where the animal was placed in the arena | • Centre (k = 64)  • Corner (k = 11) | *p* = 0.93 |
|  | 3 | Analysis software | • ANY-MAZE (k = 34)  • Ethovision (k = 14)  • TopScan (k = 12) | *p* = 0.06 |
| **Mouse analgesic drug treatment** | 4 | Height of the wall of the open field arena | • 30 cm (k = 15)  • 30.5 cm (k = 11) | *p* = 0.99 |
|  | 4 | Median age at the start of experiments | • 49 days (k = 17)  • 70 days (k = 11) | *p* = 0.52 |

***Dataset 1: effects of the experimental conditions and apparatus on thigmotactic outcome in rat modelling experiments***

Data collected for each variable for this dataset can be found in <http://osf.io/b54ra>. Overall, regressions were not statistically significant, and the regression models did not explain a substantial amount of variability observed in the target variables.

In total, 6 colours were reported for the testing arena wall, of which only black (k = 18) and transparent (k = 12) had enough comparisons for inclusion in a meta-regression (Black_estimate_ = -1.77, Transparent_estimate_ = -1.78, df = 28, *p* = 0.99, R^2^ = 0.00%) (Figure 1A).

The animal was placed either in the centre (k = 51) or at the corner (k = 12) (Centre_estimate_ = -3.61, Corner_estimate_ = -2.78, df = 61, *p* = 0.48, R^2^ = 0.00%) (Figure 1B).

The assessment duration range from 3 minutes to 60 minutes, with a median of 10 minutes. There are sufficient number of cohort-level comparisons only for the OFTs that lasted for 5 (k = 37), 15 (k = 29) and 10 (k = 14) (Coefficient = -1.13, df = 78, *p* = 0.13, R^2^ = 1.63%) (Figure 1C).

**A**

Black_estimate_ = -1.77

Transparent_estimate_ = -1.78

df = 28, *p* = 0.99

R^2^ = 0.00%

**B**

Centre_estimate_ = -3.61

Corner_estimate_ = -2.78

df = 61, *p* = 0.48

R^2^ = 0.00%

**C**

Coefficient = -0.13

df = 78, *p* = 0.13

R^2^ = 1.63%

Figure 1. Meta-regression scatterplots of the effects of (A) colour of the testing arena wall, (B) where the animal was placed in the arena and (C) the OFT duration, in rat modelling experiments. The size of the bubble represents the weight, which reflects the contribution of each comparison to the pooled effect estimate.

***Dataset 2: effects of the experimental conditions and apparatus on thigmotactic outcome in rat analgesic drug treatment experiments***

Data collected for each variable for this dataset can be found in <https://osf.io/xqgsc>. Overall, regression was not statistically significant, and the regression model only explained 1.99% of variability observed in the target variable.

The heights reported ranged from 15cm to 63cm, with a median of 45cm. Only the thigmotactic data that were assessed using a testing arena with a height of 50*cm* (k = 15) and 40*cm* (k = 14) were included in the analysis (Coefficient = 0.11, df = 27, *p* = 0.21, R^2^ = 1.99%) (Figure 2).

Coefficient = 0.11

df = 27, *p* = 0.21

R^2^ = 1.99%

Figure 2. Meta-regression scatterplots of the effect of height of the arena on thigmotaxis in rat drug treatment experiments. The size of the bubble represents the weight, which reflects the contribution of each comparison to the pooled effect estimate.

***Dataset 3: effects of the experimental conditions and apparatus on thigmotactic outcome in mouse modelling experiments***

Data collected for each variable for this dataset can be found in <https://osf.io/4m2f8>. Regression was only statistically significant for the total area of the inner zone and the regression model explained 15.35% of variability observed in this variable.

The height range from 18*cm* to 60*cm*, with a median of 40*cm*. Only the thigmotactic data that were assessed using a testing arena with a height of 40*cm* (k = 40) and 30*cm* (k = 11) were included in a meta-regression (Coeffcient = 0.18, df = 49, *p* = 0.09, R^2^ = 3.81%) (Figure 3A).

The total area of the open arena range from 529.92*cm^2^* to 33280*cm^2^*, with a median of 1600*cm^2^*. There are sufficient number of cohort-level comparisons only for a total area of 1600*cm^2^* (k = 53) and 2500*cm^2^* (k = 27) (Coefficient = -0.0012, df = 78, *p* = 0.15, R^2^ = 1.27%) (Figure 3B).

The total area of the inner zone range from 100*cm^2^* to 4275*cm^2^*, with a median of 529*cm^2^*. There are sufficient number of cohort-level comparisons only for a total area of 1296*cm^2^* (k = 24) and 400*cm^2^* (k = 22) (Coefficient = 0.0028, df = 44, *p* = 0.004, R^2^ = 15.35%) (Figure 3C).

The animal was placed either in the centre (k = 64) or at the corner (k = 11) (Centre_estimate_ = -1.47, Corner_estimate_ = -1.56, df = 73, *p* = 0.93, R^2^ = 0.00%) (Figure 3D).

In total, 12 analysis tools were reported, of which only ANY-MAZE (k = 34), Ethovision (k = 14) and TopScan (k = 12) are the eligible software to be included in a meta-regression (ANY-MAZE_estimate_ = 0.23, Ethovision_estimate_ = -0.87, TopScan_estimate_ = -2.10, df = 57, *p* = 0.06, R^2^ = 6.73%) (Figure 4).

**A**

Coefficient = 0.18

df = 49, *p* = 0.09

R^2^ = 3.81%

**B**

Coefficient = -0.0012

df = 478 *p* = 0.15

R^2^ = 1.27%

**C**

Coefficient = 0.0028

df = 44 *p* = 0.004

R^2^ = 15.35%

Centre_estimate_ = -1.47

Corner_estimate_ = -1.56

df = 73 *p* = 0.93

R^2^ = 0.00%

**D**

Figure 3. Meta-regression scatterplots of the effects of (A) height of the arena wall, (B) total area of the open arena (C) total area of the inner zone, and (D) location of where the animal was placed on thigmotaxis in mouse modelling experiments. The size of the bubble represents the weight, which reflects the contribution of each comparison to the pooled effect estimate.

**A**

**B**

**C**

ANY-MAZE_estimate_ = 0.23

Ethovision_estimate_ = -0.87

TopScan_estimate_ = -2.10

df = 57 *p* = 0.06

R^2^ = 6.73%

Figure 4. Meta-regression scatterplots of the effect of using different types of analysis tool on thigmotaxis in mouse modelling experiments. The size of the bubble represents the weight, which reflects the contribution of each comparison to the pooled effect estimate.

***Dataset 4: effects of the experimental conditions and apparatus on thigmotactic outcome in mouse analgesic drug treatment experiments***

Data collected for each variable for this dataset can be found on <https://osf.io/k64qm>. Regressions were not statistically significant, and the regression models did not explain any variability observed in the target variables.

The height range from 25*cm* to 50*cm*, with a median of 30.5*cm*. Only the thigmotactic data that were assessed using a testing arena with a height of 30*cm* (k = 15) and 30.5*cm* (k = 11) were included in a meta-regression (Coefficient = -0.018, df = 24, *p* = 0.99, R^2^ = 0.00%) (Figure 5A).

The median age range from 33.5 days to 120 days with a median of 49 days. Only the thigmotactic data that were assessed using mice with a median age of 49 days (k = 17) and 70 days (k = 11) were included in a meta-regression (Coefficient = 0.015, df = 26, *p* = 0.52, R^2^ = 0.00%) (Figure 5B).

Coefficient = -0.018

df = 24 *p* = 0.99

R^2^ = 0.00%

**B**

**A**

Coefficient = 0.015

df = 26 *p* = 0.52

R^2^ = 0.00%

Figure 5. Meta-regression scatterplots of the effect of (A) height of the arena and (B) median age on thigmotaxis in mouse drug treatment experiments. The size of the bubble represents the weight, which reflects the contribution of each comparison to the pooled effect estimate.

**Table S3.2. Correlations between thigmotaxis and the type of stimulus-evoked limb withdrawal**

| **Experiment Type** | **Figure No.** | **Thigmotactic metric** | **Type of stimulus** | **No. of k** | **Coefficient** | **Degree of freedom** | ***p* value** |
| --- | --- | --- | --- | --- | --- | --- | --- |
| **Rat modelling** | 23A | Time in the centre | Mechanical | k = 27 | 0.35 | 25 | 0.07 |
|  | 24 | Time in the centre | Cold | k = 12 | 0.37 | 10 | 0.24 |
| **Rat analgesic drug treatment** | 25A | Time in the centre | Mechanical | k = 44 | 0.50 | 42 | 0.0006 |
|  | 26 | Time in the centre | Heat | k = 11 | 0.038 | 9 | 0.91 |
| **Mouse modelling** | 27A | Time in the centre | Mechanical | k = 24 | 0.56 | 22 | 0.004 |
|  | 28 | Time in the centre | Heat | k = 12 | 0.53 | 10 | 0.08 |

**Table S3.3 Correlations between thigmotaxis and the type of stimulus-evoked limb withdrawal in rodent species that were modelled with nerve injury induced neuropathy**

| **Experiment Type** | **Figure No.** | **Thigmotactic metric** | **Type of stimulus** | **No. of k** | **Coefficient** | **Degree of freedom** | ***p* value** |
| --- | --- | --- | --- | --- | --- | --- | --- |
| **Rat modelling** | 23B | Time in the centre | Mechanical | k = 16 | 0.63 | 14 | 0.009 |
| **Rat analgesic drug treatment** | 25B | Time in the centre | Mechanical | k = 28 | 0.70 | 26 | <0.0001 |
| **Mouse modelling** | 27B | Time in the centre | Mechanical | k = 12 | 0.33 | 10 | 0.29 |

**B**

**A**

Figure 6. A Pearson’s Correlation test between time in the centre and mechanical induced behavioural outcomes in (A) rat modelling experiments (no correlation; Coefficient = 0.35, df = 25, *p* = 0.07, k = 27), and in (B) rats modelled with nerve injury neuropathy (a strong positive correlation; Coefficient = 0.63, df = 14, *p* = 0.009, k = 16). A line of best fit (in red) was drawn. SMD, standardised mean difference.

Figure 7. A Pearson’s Correlation test between time in the centre and cold induced behavioural outcomes in rat modelling experiments (no correlation; Coefficient = 0.37, df = 10, *p* = 0.24, k = 12). A line of best fit (in red) was drawn. SMD, standardised mean difference.

**B**

**A**

Figure 8. A Pearson’s Correlation test between time in the centre and mechanical induced behavioural outcomes in (A) rat drug treatment experiments (a moderate positive correlation; Coefficient = 0.50, df = 42, *p* = 0.0006, k = 44), and in (B) rats modelled with nerve injury neuropathy (a strong positive correlation; Coefficient = 0.70, df = 26, *p* <0.0001, k = 28). A line of best fit (in red) was drawn. SMD, standardised mean difference.

Figure 9. A Pearson’s Correlation test between time in the centre and heat induced behavioural outcomes in rat drug treatment experiments (no correlation; Coefficient = 0.038, df = 9, *p* = 0.91, k = 11). A line of best fit (in red) was drawn. SMD, standardised mean difference.

**A**

**B**

Figure 10. A Pearson’s Correlation test between time in the centre and mechanical induced behavioural outcomes in (A) mouse modelling experiments (a moderate positive correlation; Coefficient = 0.56, df = 22, *p* = 0.004, k = 24), and in (B) mice modelled with nerve injury neuropathy (no correlation; Coefficient = 0.33, df = 10, *p* = 0.29, k = 12). A line of best fit (in red) was drawn. SMD, standardised mean difference.

Figure 11. A Pearson’s Correlation test between time in the centre and heat induced behavioural outcomes in mouse modelling experiments (no correlation; Coefficient = 0.53, df = 10, *p* = 0.08, k = 12). A line of best fit (in red) was drawn. SMD, standardised mean difference.
