# Supplementary material for: A systematic review and meta-analysis of thigmotactic behaviour in the open field test in rodent models associated with persistent pain
Source: PLoS One. 2023 Sep 8;18(9):e0290382. doi: 10.1371/journal.pone.0290382 (PMC10490990; doi:10.1371/journal.pone.0290382)
Supplement: S5 File — (DOCX) [file pone.0290382.s005.docx]

# S5: Search Strategy and Data Extraction Criteria

1. **Search Strategy for each online database:**

***PubMed/Medline***

| **No.** | **Component** | **Terms** |
| --- | --- | --- |
| 1 | Thigmotaxis | Thigmotaxis OR thigmotactic OR open field OR anxiety OR fear |
| AND | AND | AND |
| 2 | Disease conditions | Spinal cord injury OR nerve injury or nerve injuries OR nerve transection OR nerve ligation OR neuropathy OR peripheral neuropathy OR polyneuropathy OR neuropathic OR headache OR headache-like OR migraine OR migraine like OR arthritis OR osteoarthritis OR rheumatoid arthritis OR colitis |
|  | AND |  |
| 3 | Sensory phenotypes | Pain OR hyperalgesia OR analgesia OR analgesic OR analgesics OR allodynia OR neuralgia OR hypersensitivity OR hyperalgesic OR hyperalgesia OR antinociception OR anti-nociception OR hypoalgesia OR hypoalgesic OR antihyperalgesia OR antihyperalgesia OR antihyperalgesic OR anti-hyperalgesic OR anti-allodynic OR antiallodynic OR anti-allodynia OR antiallodynia |
| AND | AND | AND |
| 4 | Rodent filters | Rodentia OR rodent OR rodents OR rat OR rats OR rattus OR norvegicus OR mouse OR mice OR murinae OR muridae OR murine OR mus OR musculus OR woodmouse OR apodemus |

***Embase (via Ovid)***

| **No.** | **Component** | **Terms** |
| --- | --- | --- |
| 1 | Thigmotaxis | Thigmotaxis OR thigmotactic OR open field OR anxiety OR fear |
| AND | AND | AND |
| 2 | Disease Conditions | Spinal cord injury OR nerve injury or nerve injuries OR nerve transection OR nerve ligation OR neuropathy OR peripheral neuropathy OR polyneuropathy OR neuropathic OR headache OR headache-like OR migraine OR migraine like OR arthritis OR osteoarthritis OR rheumatoid arthritis OR colitis |
| 3 | AND |  |
|  | Sensory phenotypes | Pain OR hyperalgesia OR analgesia OR analgesic OR analgesics OR allodynia OR neuralgia OR hypersensitivity OR hyperalgesic OR hyperalgesia OR antinociception OR anti-nociception OR hypoalgesia OR hypoalgesic OR antihyperalgesia OR antihyperalgesia OR antihyperalgesic OR anti-hyperalgesic OR anti-allodynic OR antiallodynic OR anti-allodynia OR antiallodynia |
| AND | AND | AND |
| 4 | Animal filters | Rodentia OR rodent OR rodents OR rat OR rats OR rattus OR norvegicus OR mouse OR mice OR murinae OR muridae OR murine OR mus OR musculus OR woodmouse OR apodemus |
| 5 | Limit to | (Article OR article in press OR conference abstract OR conference paper OR erratum) AND (conference proceeding OR journal OR report) |

***Web of Science***

| **No.** | **Component** | **Terms** |
| --- | --- | --- |
| 1 | Thigmotaxis | ALL = (Thigmotaxis OR thigmotactic OR open field OR anxiety OR fear) |
| AND | AND | AND |
| 2 | Disease conditions | ALL = (Spinal cord injury OR nerve injury or nerve injuries OR nerve transection OR nerve ligation OR neuropathy OR peripheral neuropathy OR polyneuropathy OR neuropathic OR headache OR headache-like OR migraine OR migraine like OR arthritis OR osteoarthritis OR rheumatoid arthritis OR colitis) |
| AND | AND | AND |
| 3 | Sensory phenotypes | ALL = (Pain OR hyperalgesia OR analgesia OR analgesic OR analgesics OR allodynia OR neuralgia OR hypersensitivity OR hyperalgesic OR hyperalgesia OR antinociception OR anti-nociception OR hypoalgesia OR hypoalgesic OR antihyperalgesia OR antihyperalgesia OR antihyperalgesic OR anti-hyperalgesic OR anti-allodynic OR antiallodynic OR anti-allodynia OR antiallodynia) |
| AND | AND | AND |
| 4 | Animal filters | ALL = (Rodentia OR rodent OR rodents OR rat OR rats OR rattus OR norvegicus OR mouse OR mice OR murinae OR muridae OR murine OR mus OR musculus OR woodmouse OR apodemus) |
| 5 | Exclude | Review OR editorial material OR book chapter |

1. **Tables 1 and 2 list in detail the information extracted from the included studies.**

**Table 5.1.** Study-level data extracted from each included study.

| **Study-level** | |
| --- | --- |
| Bibliographic detail | - First author - Year of publication - Title |
| Reporting quality | Reporting guidelines, such as the ARRIVE, were developed for the purpose to improve the reporting of animal research. The following items were extracted:   - Reference following a reporting guideline for *in vivo* experimentation - Provide evidence of reporting in accordance with the chosen guideline |
| Acclimatisation and animal husbandry | - Time period of acclimatisation to housing environment following transportation - Housing condition (i.e. presence of other animal species and/or sex in the room, number of animals per cage, temperature, humidity, noise, vibration) - Cage condition (i.e. reporting of cage size, cage floor condition) - Light-dark cycle - Type of diet |
| OFT characteristics | - Size and shape of the open field area (i.e. length, height, width and total area) - Size and shape of the inner zone (i.e. length, width and total area) - Colour of the test arena wall - Experimental environment (i.e. light intensity, temperature, humidity, noise, vibration) - Location of where animal was placed at the start of the test - Method of measurement (i.e. automated or manual) - Manufacturer of the recording camera and type of the analysis software - Was the test conducted in an isolated chamber? If not, whether other animals or the human investigator was presented in the test room? |

**Table 5.2.** Experiment-level data extracted from each included study.

| **Experiment-level** | |
| --- | --- |
| Animal | - Species - Strain - Sex - Animal supplier - Age (at the start of experiments) - Weight (at the start of experiments) |
| Disease model | - Method of model induction - Perioperative analgesic(s) given before/during/after model induction |
| Intervention | - Dose - Route of administration - Number of administrations - Time between drug treatment and model induction - Time between drug treatment and burrowing assessment |
| Outcome measure assessment | Primary outcome – thigmotaxis and total distance travelled in the OFT   - Habituation time to the test arena - Assessment duration - Direction of effect - Number of trials and time separation between trials - Time between the model induction and the first OFT - Time between the model induction and the last OFT - Time between the first treatment and the first OFT   Secondary outcome – any stimulus-evoked limb withdrawal behavioural outcomes assessed in the same cohort of animals used in the OFT   - Type of nociceptive assessment |
| Numerical outcome data | - Unit - Mean outcome - Variance - Number of animals per group - Number of groups served by control group |

**Table 5.3. Experimental design variables relating experimental conditions and OF apparatus**.

| Experimental conditions | - OFT conducted in the light or dark phase - The presence of other animal subjects in the testing room during the OFT - The presence of the human investigator in the testing room during the OFT - Test arena light intensity - Temperature of the testing room - Humidity of the testing room - Noise level in the testing room - Vibration level in the testing room - Habituation time prior to the OFT - Assessment duration of the OFT - Location of where the animal was placed in the arena at the start of the OFT - Number of OFT trials conducted in total after model induction - Time between model induction and the first OFT assessment - Time between model induction and the last OFT assessment - Age of the animals - Weight of the animals |
| --- | --- |
| OF apparatus | - Shape of the inner zone - Shape of the open arena - Height of the open arena - Total area of the inner zone - Total area of the open arena - Colour of the testing arena wall - Method of the measurement (i.e. automated or manual) - If automated, the analysis software that was used - Manufacturer of the recording camera that was used |
