# Supplementary material for: A systematic review and meta-analysis of thigmotactic behaviour in the open field test in rodent models associated with persistent pain
Source: PLoS One. 2023 Sep 8;18(9):e0290382. doi: 10.1371/journal.pone.0290382 (PMC10490990; doi:10.1371/journal.pone.0290382)
Supplement: Attachment — Submitted filename: Response to Reviewers.docx [file pone.0290382.s007.docx]

Pain Research

Department of Surgery and Cancer

Imperial College London

Chelsea and Westminster Hospital

369 Fulham Road

London

SW10 9NH

Tel: +44(0)7793708361

[n.soliman16@imperial.ac.uk](mailto:n.soliman16@imperial.ac.uk)

29 June 2023

Editor-in-Chief

Dr Emily Chenette

Dear Dr Chenette,

Thank you for the reviews of our manuscript titled, ‘A systematic review and meta-analysis of thigmotactic behaviour in the open field test in rodent models associated with persistent pain’ which we submitted for your consideration in *PLOS One*.

We enclose a revised manuscript that addresses all comments raised by the editor and reviewer as detailed in our response below. We thank the editor and reviewer for their positive, insightful and constructive feedback. We agree with the points raised and have incorporated the suggestions as best as possible. This has included some of the data tables and figures being relocated to supplementary files. We feel that these revisions have significantly improved the overall quality of the manuscript, which we hope will now be accepted for publication in *PLOS One*.

I would like to amend the funding statement and financial disclosure to read, “XYZ was in receipt of funding from the Medical Research Council (grant number MR/N014103/1) and NS funded by the Jennie Gwynn Legacy Fund. The funders had no role in study design, data collection and analysis, decision to publish, or preparation of the manuscript. The remaining author received no specific funding for this work.”

All authors have contributed to this systematic review and meta-analysis and approved it for submission. It is not being considered by another journal. The data has been shared as supplementary digital content and is linked to the project repository on the Open Science Framework (DOI:10.17605/OSF.IO/RMT97).

Thank you for your consideration and we look forward to receiving your decision due course.

Yours sincerely,

Nadia Soliman, PhD

ACADEMIC EDITOR:

- please do follow the suggestion provided by reviewers

- have in mind that some of the data could be relocated to supplementary files.

- do support your study introduction and discussion with relevant and the most recent reviews and studies

We have moved from the results section Tables 3, 4, 5, 6 and Figures 23-28 to supplementary file, S3. We have also moved Tables 8, 9, and 11 to supplementary file, S5 from the methods section. This reduced the number of tables by 7 from 11 to 4, and the figures by 4, from 32 to 28. Please see below for our response to the reviewer and incorporation of their suggestions.

Reviewer #1: This manuscript by Zhang and colleagues is a systematic review and meta-analysis of thigmotaxis in models of pain. I am impressed by the still rather novel attempt to meta-analyze preclinical data, although this paper again shows why it doesn’t really show much: simply not enough variability in methods (e.g., use of different sexes, strains, multiple drugs). I’m also a bit underwhelmed by the stakes here; 30+ figures and long, overly detailed tables in service of what most people in the field view as a rather secondary issue (pain comorbidities, or potential confounds). That said, I realize PLoS One is not concerned about how important this all is, but rather whether it’s solid. It clearly is. This group is absolutely at the forefront of preclinical pain meta-analysis. I have two major pieces of advice to improve things.

We share the reviewer’s underwhelm and agree that this review hasn’t led to the insights one would hope for to progress the field. There remains an ethical and societal obligation to continue to examine the use of animal models in pain research and given the volume and exponential production of data, systematic reviews are likely to play an important role. However, the lack of variability in experimental designs and the paucity of reporting experimental details are limiting. We know pain studies are affected by a range of factors including methodological rigour, animal characteristics, experimental paradigms and the environment, however, as this review demonstrates, we are yet to see the changes in the field that would improve our appreciation of these factors.

The first is that to my mind the group failed to analyze what I think is probably the most important issue: time. They conclude that thigmotaxis is caused by persistent pain. Um, duh. We knew this. The outstanding question in the field is how long does it take for the anxiety (which is driving the thigmotaxis) to develop after the injury. This information (time of anxiety testing post-injury) is in every paper they analyzed, but I see no evidence that it was looked at here.

We collected time from model intervention to open field test and have conducted an analysis of time in rat and mouse nerve injury models. The methods, results and discussion have been changed to reflect this. The analysis was performed on rat and mouse nerve injury models (the most frequently reported) and are presented in figures 7 and 20.

Results section 2.3.1., *“Open field tests were conducted from immediately after model induction to 35 days after nerve injury (for which there were 41 comparisons that reported time since model induction). Time accounted for a significant proportion of heterogeneity (Q = 652.50; d.f. = 13; p <0.0001) (Figure 7A). However, this was not the case when time was analysed in weekly blocks (Q = 3.31; d.f. = 3; p =0.35) (Figure 7B).”*

Results section 2.3.2., *“Open field tests were conducted between 24 h and 180 days after nerve injury (for which there were 46 comparisons that reported time since model induction). All time points accounted for a significant proportion of heterogeneity (Q = 634.39; d.f. = 19; p <0.0001) (Figure 20A). The same was observed in the time block analysis (Q = 16.61; d.f. = 3; p =0.0009) and the 2-3 week time block resulted in the largest observed effect sizes (Figure 20B). However, these findings should be interpreted with caution given the low number of comparisons for each time period.”*

Discussion, *“Given the broad range of timings that the OFTs were conducted and limited comparisons within each period, we were also unable to discern how long after model induction it takes for thigmotactic behaviour to develop.”*

The second is that I don’t like the introduction at all. The citations are utterly arbitrary, in some cases randomly picking a few papers when much better reviews exist, and in others using arbitrarily chosen reviews instead of citing primary literature proving a point.

The introduction has been updated. We have included a broader range of more recent and relevant primary studies and reviews. This includes references to the systematic reviews Currie et al., 2019, Soliman et al., 2021 and narrative reviews Sadler et al., 2022, and Tappe-Theodor et al., 2014.

But the bigger problem is that the discussion is about predictive validity of outcome measures of pain, when the paper is actually meta-analyzing not an outcome measure of pain, but rather a measure of a pain-induced comorbidity: anxiety. No one in the preclinical pain field is pretending that thigmotaxis is a measure of pain itself, however indirect. They are using it specifically to address the presence or absence of the comorbidity. Thus, I think this paper would be a lot better set up by a discussion of the prevalence of pain comorbidities and their modelling in animals.

We have described thigmotaxis as a behaviour not a measure of pain. Pain cannot be directly measured in humans or animals. We have contextualised thigmotaxis to pain-associated models and whether thigmotactic behaviour can be attenuated by analgesic drugs, thereby distinguishing between pain and anxiety. We appreciate that thigmotaxis can be influenced by other co-morbidities and this was discussed within the introduction e.g., *“This behavioural paradigm is commonly used for animal research of psychiatric disorders, particularly anxiety…”* and “*It could also reflect the clinical observations of exacerbated avoiding behaviours and anxiodepressive disorders that are associated with patients with chronic pain”.* Measuring an ethologically relevant behaviour allows us to measure the global affect of a pain-associated intervention. The following sentence has been added to the introduction to further emphasise the points made by the reviewer, *“It has been postulated that the behaviour could give insight into the affective, cognitive and sensory dysfunction associated with pain.”*
